# Targeted exome sequencing for molecular diagnosis of pediatric Alport syndrome in Southwest China

**DOI:** 10.3389/fgene.2025.1580864

**Published:** 2025-08-29

**Authors:** Cong Zhou, Yuanyuan Xiao, Xing Wei, Jing Wang, Shanling Liu

**Affiliations:** ^1^ Department of Medical Genetics / Prenatal Diagnostic Center, West China Second University Hospital, Sichuan University, Chengdu, China; ^2^ Key Laboratory of Birth Defects and Related Diseases of Women and Children (Sichuan University), Ministry of Education, Chengdu, China

**Keywords:** Alport syndrome, *COL4A3*, *COL4A4*, *COL4A5*, variants, targeted exome sequencing

## Abstract

**Background:**

Alport syndrome (AS) is an inherited disorder affecting basement membrane collagen IV. AS is characterized by hematuria and progressive renal failure, accompanied by high-frequency sensorineural deafness and ocular changes. AS is caused by collagen type IV α3 chain (*COL4A3*), α4 chain (*COL4A4*), and α5 chain (*COL4A5*) variants. We aimed to identify the genetic variants in a cohort of 20 children with clinically suspected AS in Southwest China.

**Results:**

We detected 21 *COL4A3*, *COL4A4*, and *COL4A5* variants in 20 probands. Of these variants, 16 (16/21, 76.2%) were classified as pathogenic/likely pathogenic and 5 (5/21, 23.8%) of uncertain significance according to the American College of Medical Genetics and Genomics criteria. A total of 11 (11/21, 52.4%) and 10 (10/21, 47.6%) variants were known and novel, respectively.

**Conclusion:**

We performed molecular diagnosis on 15 patients using targeted exome sequencing. Our findings indicate additional *COL4A3*, *COL4A4*, and *COL4A5* variants as involved in AS, having implications for genetic diagnosis, therapy, and counseling of affected families.

## Introduction

Alport syndrome (AS) is the second-most common renal monogenic disorder after autosomal-dominant polycystic kidney disease (Nozu et al., 2019). AS is an inherited disorder affecting basement membrane collagen IV, characterized by hematuria and progressive renal failure and accompanied by high-frequency sensorineural deafness and ocular changes (Kashtan, 2021; Kashtan and Gross, 2021). AS is caused by *COL4A3* (OMIM:120070), *COL4A4* (OMIM:120131), and *COL4A5* (OMIM:303630) variants (Nozu et al., 2019; Torra et al., 2025). These cause functional impairment of the glomerular basement membrane (GBM) in the kidney (Kashtan, 2021; Kashtan and Gross, 2021). *COL4A3* and *COL4A4* variants encoding the collagen type IV α3 and α4 chains are responsible for autosomal-recessive and autosomal-dominant AS. *COL4A5* variants encoding the collagen type IV α5 chain are responsible for X-linked AS, which is typically more severe in men (Savige et al., 2015; Nozu et al., 2019; Kashtan, 2021; Kashtan and Gross, 2021; Gregorio et al., 2023; Torra et al., 2025). AS prevalence ranges from 1/5,000 to 1/53,000 of the population. Approximately 80%–85%, 15%, and <5% of the AS cases are X-linked, autosomal-recessive, and autosomal-dominant AS, respectively (Savige et al., 2021).

The onset of AS symptoms usually occurs the first decade of life but may not be detected (Savige et al., 2013; Gross et al., 2020; Kashtan, 2021; Kashtan and Gross, 2021). The clinical diagnoses of AS and renal diseases can be easily confused, resulting in misdiagnosis or missed diagnosis (Savige et al., 2015; Nozu et al., 2019; Kashtan, 2021; Kashtan and Gross, 2021). The prognosis for AS is poor, and most children with AS progress to renal failure (Nozu et al., 2019; Gross et al., 2020). The indicators available for monitoring AS are limited to proteinuria and renal function and no curative therapy is currently available for it. Angiotensin-converting enzyme inhibitors delay ESRD (Torra and Furlano, 2019; Pedrosa et al., 2021). Therefore, the early and definite diagnosis of AS in children is crucial for commencing treatment, predicting kidney prognosis, and providing genetic counseling (Kashtan et al., 2021).

Genetic testing is a tool for definitively diagnosing and determining the prognosis of AS, with results supporting the provision of counseling to patients and their family members. Next-generation sequencing (NGS) is a high-throughput sequencing method used to rapidly sequence the exome, transcriptome, and genome (Adams and Eng, 2018). Expert guidelines recommend genetic testing for AS diagnosis, and genes where mutations produce clinical features similar to AS should be examined (Savige et al., 2019). Targeted exome sequencing was performed on 20 Chinese children with suspected AS in this study. Ten novel and eleven known *COL4A3*, *COL4A4*, and *COL4A5* variants were identified.

## Methods

### Patients

A total of 20 children from 20 unrelated families with one or more members experiencing hematuria or extrarenal symptoms as initial clinical impression were included. The children were referred to the West China Second University Hospital, Sichuan University, for genetic diagnosis between August 2017 and January 2021. Clinical patient data were collected, and the following information was recorded for all patients: prenatal/birth history, family history, sex, age at NGS testing (enrolment), age at onset, hematuria, proteinuria, and extrarenal symptoms. The renal histopathological findings were obtained using light microscopy and electron microscopy (EM). Patients who met one of the following criteria were included in this study: ① family history of hematuria; ② proteinuria, chronic kidney disease, or renal failure; ③ negative or nonspecific renal biopsy results on routine immunofluorescence; ④ characteristic changes in basement membrane evident on EM of renal tissue sections, such as irregular thickening, thinning, and division; ⑤ abnormal staining of type IV collagen α chains in the kidney or skin; ⑥ deafness associated with chronic kidney disease; ⑦ sensorineural deafness, anterior cone lens, and/or characteristic retinopathy.

Peripheral blood samples from patients and their family members were collected for targeted exome sequencing and Sanger sequencing. Written informed consent was obtained from the parents or legal guardians before genetic testing. This study was approved by the Medical Ethics Committee of West China Second University Hospital, Sichuan University, China.

### Targeted exome sequencing and data analyses

The genomic DNA was extracted from peripheral blood leukocytes using a QIAamp DNA Blood Mini kit (Qiagen GmbH, Hilden, Germany) and stored at −20 °C until use. The relatedness between the proband and their parents was confirmed via quantitative fluorescence polymerase chain reaction (PCR) using short tandem repeats. The exomes of a disease-targeted gene panel (selected genes sequenced by NGS) were sequenced for each proband using an M167 Targeted Exome Capture kit (MyGenostics, Inc., Beijing, China) according to the manufacturer’s instructions. The kit targeted 504 genes (Supplementary Table S1) that cause renal disease (such as *COL4A3*, *COL4A4*, and *COL4A5*). A DNA library was constructed according to the instructions. The libraries of the captured genomic DNA were sequenced using the NextSeq 500 platform (Illumina, San Diego, CA, United States) to generate paired-end reads for 150 cycles per read. Adapter, low-quality, multiple-N, and short reads (<40 bp) were removed using Cutadapt. The reads were mapped to the reference human genome (GRCh37/hg19) using Burrows–Wheeler Aligner (BWA-MEM, version 0.7.10). Variants were determined using the Genome Analysis Tool Kit (version 4.0.8.1) (Han et al., 2020). Annovar (version 1) was used to annotate the variants. All sequencing datasets must satisfy the following quality control criteria: coverage of target region: ≥98%; average sequencing depth on target: ≥100×; fraction of target covered with at least 20×: ≥85%; Q30: ≥85%. All variants were filtered based on their frequency in public data. The variants with a minor allele frequency of <0.05 were retained (Zhang et al., 2020). Several variant prediction tools were used to predict the functional impact of the candidate variants. The variants were classified based the American College of Medical Genetics and Genomics (ACMG) variant interpretation guidelines (Richards et al., 2015). Finally, the variant function and the correlation of the variant with the disease phenotype were determined using the Online Mendelian Inheritance Man database.

### Sanger sequencing and real-time PCR

Sanger sequencing was used to confirm the candidate variants identified using targeted exome sequencing. The primers (Supplementary Table S2) used to amplify the fragments harboring the variants were designed using Primer Premier 5 (Premier Biosoft, San Francisco, CA, United States) and synthesized by Sangon Biotech (Shanghai, China). The Sanger sequencing data were analyzed using Chromas software (Technelysium, Australia). Real-time PCR was performed for analyzing the segregation of the fragment deletions using primers designed according to the variant sequences.

## Results

### Patient characteristics

The 20 patients originated from 12 cities across 17 distinct regions. Some patients were treated at local hospitals for many years without a definitive diagnosis and were referred to our hospital. The ages of the children ranged from 1 to 14 years (median, 6.5 years). There were eleven girls (11/20, 55%) and nine boys (9/20, 45%). The duration from symptom onset to diagnosis varied from 1 to 13 years (median, 3 years). Hematuria was a universal feature in our cohort, being present in all 20 children. Proteinuria within the non-nephrotic range was identified in 13 children (13/20, 65%). Proteinuria was undetectable in the remaining seven (7/20, 35%) children. One child (1/20, 5%) was confirmed as having moderate sensorineural hearing loss (patient 11), and one (1/20, 5%, patient 5) presented with bilateral refractive errors. A total of 15 (15/20, 75%) children had a family history of kidney disease, whereas kidney disease was sporadic in the remainder of the children.

Renal biopsies were performed for 12 (12/20, 60%) children before targeted exome sequencing. Table 1 summarizes the details of the clinical findings.

**TABLE 1:** Clinical data of patients.

Patient ID	Gender	Current age (years)	Onset age (years)	Renal pathology	Hematuria	Proteinuria	Ocular lesions	Hearing loss	Initial clinical impression	Family history
1	M	1	1	Mild mesangial proliferative glomerulopathy	Yes	Yes	Normal	Normal	Hematuria, proteinuria	P
2	F	3	1	ND	Yes	Normal	Normal	Normal	Hematuria	P
3	F	4	2	ND	Yes	Yes	Normal	Normal	Hematuria, proteinuria	P
4	M	13	12	MGA and thin basement membrane	Yes	Yes	Normal	Normal	TBME; AS?	P
5	F	6	6	Thined GBM	Yes	Normal	Bilateral refractive errors	Normal	IgA nephropathy	P
6	F	4	4	ND	Yes	Yes	Normal	Normal	Benign familial hematuria	P
7	F	2	2	ND	Yes	Normal	Normal	Normal	Hematuria	P
8	M	11	5	MGA	Yes	Yes	Normal	Normal	Nephrotic syndrome	N
9	M	6	2	Glomerular segmental mesangial proliferation	Yes	Yes	Normal	Normal	Hematuria, proteinuria	N
10	F	3	2	ND	Yes	Normal	Normal	Normal	Microscopic haematuria	P
11	M	11	9	TBMN	Yes	Normal	Normal	Moderate bilateral SNHL	Primary nephrotic syndrome	N
12	F	7	7	ND	Yes	Yes	Normal	Normal	AS?	P
13	F	6	2	TBMN	Yes	Yes	Normal	Normal	AS?	N
14	M	12	2	MGA	Yes	Yes	Normal	Normal	Hematuria, proteinuria	P
15	F	6	3	ND	Yes	Normal	Normal	Normal	Hematuria	P
16	F	8	3	MGA	Yes	Normal	Normal	Normal	IgA nephropathy?	P
17	M	9	2	Irregular GBM thinning and thickening with lamellation and focal disruptions	Yes	Yes	Normal	Normal	AS?	P
18	M	10	9	ND	Yes	Yes	Normal	Normal	Hematuria, proteinuria	P
19	F	10	9	MGA, segmental thinning of GBM with lamellation and focal disruptions	Yes	Yes	Normal	Normal	AS	N
20	M	14	13	Irregular GBM thickening with lamina densa accentuation, exhibiting focal basket-weave lamellation and microtearing. Podocyte foot processes show segmental effacement	Yes	Yes	Normal	Normal	AS	P

Male (M); female (F); not detected (ND); minor glomerular abnormalities (MGA); thin basement membrane nephropathy (TBMN); sensorineural hearing loss (SNHL); glomerular basement membrane (GBM); Alport syndrome (AS); negative (N); positive (P).

### Gene analysis

We detected 21 *COL4A3*, *COL4A4*, and *COL4A5* variants in the 20 probands. Table 2 details the variants of these genes (patient 2 harbored two variants; the remaining children each carried one variant). Overall, we identified ten, five, two, two, and two missense, frameshift, nonsense, deletion, and splice variants, respectively. We classified 16 (16/21, 76.2%) variants as pathogenic/likely pathogenic according to the ACMG criteria (Richards et al., 2015). The remaining five (5/21, 23.8%) variants were classified as of uncertain significance. A total of eleven (11/21, 52.4%) and ten (10/21, 47.6%) were known and novel, respectively. Two of the novel variants were located in *COL4A3:* c.871G>A and c.1908dup, two were identified in *COL4A4:* c.2377dup and c.3230del, and six were located in *COL4A5:* c.1033-1G>C, c.1951_1954dup, c.2403del, c.2909G>A, c.3833G>T, and c.4730A>C. All variants, except for the exon14_19 variant in *COL4A5* in patient 20, were confirmed via Sanger sequencing of the patients and their family members. All gene variants in the family members (including parents as well as paternal and maternal grandparents) indicated co-separation (Supplementary Table S3). In addition, ten (10/21, 47.6%) and five (5/21, 23.8%) were maternally and paternally inherited, respectively; five of the 21 (5/21, 23.8%) were *de novo* variants. Testing of family members was refused for one child (patient 20).

**TABLE 2:** Summary of variants identified in the patients.

Patient ID	Gene	Transcript accession no.	Exon/Intron	Nucleotide change	Amino acid change	Status	Variant type	Segregation tested	Inheritance	ACMG classification	References
1	*COL4A3*	NM_000091	Exon15	c.871G>A	p.G291R	Het	Missense	Yes	Father	LP (PM2_Supporting, PP1, PP3_Strong)	This study
2	*COL4A3*	NM_000091	Exon16	c.898G>A	p.G300R	Het	Missense	Yes	Father	LP (PS4_Supporting, PM2_Supporting, PP3_Strong)	Mencarelli et al. (2015)
2	*COL4A3*	NM_000091	Exon19	c.1038T>A	p.Y346*	Het	Nonsense	Yes	Mother	LP (PVS1, PM2_Supporting)	Zhang et al. (2021)
3	*COL4A3*	NM_000091	Exon26	c.1908dup	p.G637Rfs*9	Het	Frameshift	Yes	Mother	LP (PVS1, PM2_Supporting)	This study
4	*COL4A3*	NM_000091	Exon38	c.3321_3329del	p.S1108_G1110del	Het	Small deletions	Yes	Mother	VUS (PM2_Supporting, PM4)	Longo et al. (2002)
5	*COL4A4*	NM_000092	Exon28	c.2377dup	p.A793Gfs*2	Het	Frameshift	Yes	Father	P (PVS1, PM2_Supporting, PP1)	This study
6	*COL4A4*	NM_000092	Exon35	c.3230del	p.P1077Lfs*64	Het	Frameshift	Yes	Mother	P (PVS1, PM2_Supporting, PP1)	This study
7	*COL4A4*	NM_000092	Exon46	c.4423G>T	p.D1475Y	Het	Missense	Yes	Father	VUS (PM2_Supporting)	Carmignac et al. (2012)
8	*COL4A5*	NM_000495	Exon15	c.871C>T	p.Q291*	Hemi	Nonsense	Yes	*De novo*	P (PVS1, PS2_Moderate, PM2_Supporting)	Yamamura et al. (2017)
9	*COL4A5*	NM_000495	Intron18	c.1033-1G>C	Splicing	Hemi	Splicing	Yes	*De novo*	P (PVS1, PS2_Moderate, PM2_Supporting)	This study
10	*COL4A5*	NM_000495	Exon24	c.1672G>A	p.G558S	Het	Missense	Yes	Father	LP (PS4_Supporting, PM1, PM2_Supporting, PP1, PP3_Moderate)	Di et al. (2022)
11	*COL4A5*	NM_000495	Exon26	c.1951_1954dup	p.K652Tfs*2	Hemi	Frameshift	Yes	*De novo*	P (PVS1, PS2_Moderate, PM2_Supporting)	This study
12	*COL4A5*	NM_000495	Exon28	c.2215C>G	p.P739A	Het	Missense	Yes	Mother	VUS (PM2_Supporting)	Wang et al. (2012)
13	*COL4A5*	NM_000495	Exon30	c.2403del	p.G802Dfs*17	Het	Frameshift	Yes	*De novo*	P (PVS1, PS2_Moderate, PM2_Supporting)	This study
14	*COL4A5*	NM_000495	Exon32	c.2678G>T	p.G893V	Hemi	Missense	Yes	Mother	LP (PS4_Supporting, PM2_Supporting, PP3_Strong)	Wang et al. (2012)
15	*COL4A5*	NM_000495	Exon33	c.2909G>A	p.G970D	Het	Missense	Yes	Mother	VUS (PM2_Supporting, PP1, PP3_Moderate)	This study
16	*COL4A5*	NM_000495	Intron38	c.3454 + 1G>C	Splicing	Het	Splicing	Yes	Mother	LP (PVS1, PM2_Supporting)	Netzer et al. (1993)
17	*COL4A5*	NM_000495	Exon39	c.3544G>C	p.G1182R	Hemi	Missense	Yes	Mother	LP (PS4_Supporting, PM2_Supporting, PP3_Strong)	Kawai et al. (1996)
18	*COL4A5*	NM_000495	Exon42	c.3833G>T	p.G1278V	Hemi	Missense	Yes	Mother	VUS (PM2_Supporting, PP3)	This study
19	*COL4A5*	NM_000495	Exon49	c.4730A>C	p.H1577P	Het	Missense	Yes	*De novo*	LP (PS2_Moderate, PM2_Supporting, PP3_Strong)	This study
20	*COL4A5*	NM_000495	Exon14_19del	Not yet available	Not yet available	Hemi	Gross deletions	Yes	Unknown	P (PVS1, PS4_Supporting, PM2_Supporting)	Nagano et al. (2018)

heterozygous (Het); hemizygote (Hemi); American College of Medical Genetics and Genomics (ACMG); pathogenicity (P); likely pathogenicity (LP); uncertain significance (VUS).

## Discussion

AS is characterized by hematuria, proteinuria, and progressive chronic renal failure and may involve ocular and auditory abnormalities (Nozu et al., 2019; Kashtan, 2021; Kashtan and Gross, 2021). AS is a genetically heterogeneous disorder, with all forms resulting from mutations of the genes that code type IV collagen, *COL4A3*, *COL4A4,* and *COL4A5.* Type IV collagen is a major structural component of the basement membrane (Savige et al., 2015; Kashtan, 2021; Kashtan and Gross, 2021; Torra et al., 2025). The type IV collagen network is composed of COL4A3-5 chains. Type IV collagen is only expressed in the GBM and basal membrane of the distal renal tubules, alveoli, eyes, and cochlea (Nozu et al., 2019; Gregorio et al., 2023). Variants in these genes lead to the irreversible lamination and rupture of the basement membrane and eventually to progressive kidney failure, blurred vision, and sensorineural deafness (Fallerini et al., 2014). Early treatment can delay ESRD onset and prolong the life expectancy of patients with AS (Kashtan, 2021; Kashtan and Gross, 2021). Therefore, genetic testing of children with a family history or early occurrence of hematuria and proteinuria will contribute to early diagnosis and treatment, reduce complications, and decrease the risk of other infectious diseases, thus demonstrating important clinical significance (Savige et al., 2019).

Currently, there are several commonly used diagnostic methods for AS. For diagnosis based on clinical manifestations, the typical clinical manifestations of AS are the combined presence of hematuria, deafness, and a family history of renal failure (Kashtan, 2021; Kashtan and Gross, 2021). However, AS is heterogeneous and easily missed based on clinical manifestations. Diagnosis can also be made based on type IV collagen α chain staining of renal or skin tissue. Immunofluorescence staining of type IV collagen α chain of the skin basement membrane has become widely used in clinical practice in recent years. However, approximately 10%–20% of patients with AS do not show significant abnormal expression of type IV collagen α chain in the renal tissue or skin tissue of GBM, indicating that its normal expression cannot exclude AS (Hashimura et al., 2014; Nozu et al., 2019). For diagnosis based on EM of renal tissues, the typical pathological changes in patients with AS are uneven thickness of GBM, laceration, stratification, and reticulation (Savige et al., 2015; Nozu et al., 2019; Gregorio et al., 2023). However, there are also limitations in diagnosing AS based on EM. Renal biopsy is an invasive procedure and is not suitable for younger children. In addition, the results of EM cannot identify the genetic pattern, thereby failing to address the needs of patients concerning genetic counseling. The consensus statement on standards and guidelines for the molecular diagnostics of AS suggests that when AS is suspected, genetic testing should take precedence over renal biopsy for diagnosis. If a pathogenic variant is confirmed, renal biopsy may not be required (Savige et al., 2021). Genetic testing is noninvasive, has a high detection rate, and can determine the genetic pattern of AS and exclude other hereditary nephropathies. It is also an important method for prenatal diagnosis and provides the possibility for future gene therapy.

AS-related genes are *COL4A3* (52 exons in total), *COL4A4* (48 exons in total), and *COL4A5* (51 exons in total). Sanger sequencing is considered the “gold standard” for the diagnosis of genetic disease. However, it is time-consuming and laborious (Adams and Eng, 2018). In addition, it is not suitable for AS genes with several exons. In contrast, NGS is a high throughput and rapid procedure (Jacob, 2013; Pulignani et al., 2018). There are three detection methods based on NGS: whole-genome sequencing (WGS), whole-exome sequencing (WES), and targeted exome sequencing. WGS is expensive and unsuitable for patients (Adams and Eng, 2018). Data analysis of WES is complex, and most patients need to undergo trio-WES. Furthermore, the cost of WES is prohibitive for some families (Adams and Eng, 2018). Targeted exome sequencing is typically used for specific suspected diseases or a group of diseases. This sequencing has the advantages of high flux, speed, and lower detection cost per unit point. Its purpose is to maximize the coverage, sensitivity, and specificity of the contained genes, allowing for simpler, economical, and easier analysis (Jacob, 2013; Adams and Eng, 2018; Pulignani et al., 2018). Moreover, targeted exome sequencing has unique advantages in detecting deep intronic regions and somatic chimerism (Jacob, 2013; Adams and Eng, 2018; Pulignani et al., 2018).

In 2015, the Japanese Society of Pediatric Nephrology (JSPN) diagnostic criteria established by the Working Group for AS suggested that in addition to the primary feature (persistent hematuria), patients with AS should satisfy one or more secondary features (mutations in *COL4* genes, abnormal *COL4* expression, and GBM-specific abnormalities) or two or more accessory features (family history of kidney diseases, bilateral sensorineural deafness, ocular abnormalities, and diffuse leiomyomatosis) (Nakanishi and Yoshikawa, 2015; Nozu et al., 2019). Using the JSPN diagnostic criteria, 15 of the 20 children in the present study were diagnosed with AS. However, the relatively small cohort limits statistical power and the ability to detect rare variants or subtle genotype-phenotype correlations in our study. Additionally, all 20 probands originated from Southwest China, introducing potential regional sampling bias. A positive family history was present in 75% of cases, which may lead to an overestimation of hereditary AS rates. Thus, multi-center cohorts are needed to validate variant prevalence estimates, analyze the environmental influences on phenotypic modulation, and investigate the ethnicity-specificity of these novel variants.

A total of 2,633 disease-causing variants of AS have been recorded according to HGMD Professional 2025.1 (https://my.qiagendigitalinsights.com/bbp/view/hgmd/pro/start.php), as well as 498 (498/2,633, 18.9%), 482 (482/2,633, 18.3%), and 1,653 (1,653/2,633, 62.8%) disease-causing variants of *COL4A3*, *COL4A4*, and *COL4A5*, respectively. Table 3 lists the proportions of the variation types corresponding to *COL4A3*, *COL4A4*, and *COL4A5*. The majority of the variants were missense mutations (265/498, 53.2%; 230/482, 47.7%, and 697/1,653, 42.2% in *COL4A3*, *COL4A4*, and *COL4A5*, respectively). No hotspot mutations were found in the three genes. We identified 21 *COL4A3*, *COL4A4*, and *COL4A5* variants, whose pathogenicity was evaluated according to the ACMG criteria (Table 2). These included ten novel variants: eight classified as pathogenic/likely pathogenic and two variants of uncertain significance. The p.G291R variant detected in our cohort localizes to the same residue as the heterozygous likely pathogenic p.G291E variant in *COL4A3* reported by Groopman et al. (2019) in European-ancestry patients, representing a distinct amino acid substitution. Our splice variant c.1033-1G>C occurs within the same splice region as the c.1033–2A>G variant identified by Chen et al. (2021) in an affected pedigree. They demonstrated that c.1033–2A>G induces frameshift and premature termination through RNA splicing analysis in cellular models. Due to the absence of functional validation, the pathogenicity of eight novel variants remains to be confirmed. For the two families with VUS classifications, we will continue to monitor these patients and perform genotype-phenotype analyses. We also recommend variant re-evaluation or supplemental whole-exome sequencing in the future.

**TABLE 3:** Number of variants in *COL4A3*, *COL4A4*, and *COL4A5* of Alport syndrome.

Gene	*COL4A3*		*COL4A4*		*COL4A5*	
Mutation type	Numbers of variants	Ratio	Numbers of variants	Ratio	Numbers of variants	Ratio
Nonsense	35	7.0%	39	8.1%	110	6.7%
Missense	265	53.2%	230	47.7%	697	42.2%
Splicing	72	14.5%	62	12.9%	277	16.8%
Small deletions	74	14.9%	92	19.1%	274	16.6%
Small insertions	25	5.0%	29	6.0%	95	5.7%
Small indels	2	0.4%	3	0.6%	20	1.2%
Gross deletions	19	3.8%	23	4.8%	165	10.0%
Gross insertions	6	1.2%	4	0.8%	7	0.4%
Complex rearrangements	0	0	0	0	8	0.4%
Total	498	100	482	100	1,653	100

In the analyses of associations between the novel variants and phenotypic severity, we find that male patients with X-linked AS exhibited more severe clinical manifestations, and autosomal dominant AS and female X-linked AS patients demonstrated broader phenotypic variability. Furthermore, protein-truncating variant types in patients with X-linked AS present with severe symptoms more than missense variant types. These findings align with established genotype-phenotype relationships in AS literature (Gregorio et al., 2023). Identifying these previously unreported variants expands the mutational spectrum of AS-associated genes and adds valuable knowledge to the field and may stimulate further research.

## Conclusion

Genetic testing is crucial for diagnosing AS. The targeted exome sequencing of the multiple genes related to kidney disease enabled the simultaneous, rapid, cost-effective, and accurate analysis of *COL4A3*, *COL4A4*, and *COL4A5*. We utilized targeted exome sequencing to achieve molecular diagnosis in 15 patients. Our findings describe additional *COL4A3*, *COL4A4*, and *COL4A5* variants that lead to AS and have implications for genetic diagnosis, therapy, and counseling of affected families.

## Data Availability

For the considerations about the security of human genetic resources and the confidentiality of participant, the data is not publicly available, but can be applied from the corresponding author on reasonable request.

## Abbreviations

Alport syndrome (AS); collagen type IV α3 chain (COL4A3); collagen type IV α4 chain (COL4A4); collagen type IV α5 chain (COL4A5); end-stage renal disease (ESRD); glomerular basement membrane (GBM); next-generation sequencing (NGS); electron microscopy (EM); polymerase chain reaction (PCR); American College of Medical Genetics and Genomics (ACMG); Japanese Society of Pediatric Nephrology (JSPN).
