# Supplementary material for: Targeted exome sequencing for molecular diagnosis of pediatric Alport syndrome in Southwest China
Source: Front Genet. 2025 Aug 29;16:1580864. doi: 10.3389/fgene.2025.1580864 (PMC12425891; doi:10.3389/fgene.2025.1580864)
Supplement: Supplementary file 1 [file DataSheet1.docx]

Table S3. Phenotype and the variant carrying status of the patients’ family members.

| Patient ID | Gene | Nucleotide change | Father | | Mother | | (Paternal) Grandfather | | (Paternal)  Grandmother | | (Maternal) Grandfather | | (Maternal) Grandmother | |
| --- | --- | --- | --- | --- | --- | --- | --- | --- | --- | --- | --- | --- | --- | --- |
|  |  |  | S | P | S | P | S | P | S | P | S | P | S | P |
| 1 | *COL4A3* | c.871G>A | Het | Proteinuria, occult blood | W | N | Het | Occult blood | W | N | ND | N | ND | N |
| 2 | *COL4A3* | c.898G>A | Het | Occult blood | W | Nephrolithiasis | ND | N | ND | N | ND | N | ND | N |
| 2 | *COL4A3* | c.1038T>A | W | Occult blood | Het | Nephrolithiasis | ND | N | ND | N | ND | N | ND | N |
| 3 | *COL4A3* | c.1908dup | W | N | Het | Microalbumin | ND | N | ND | N | ND | N | ND | N |
| 4 | *COL4A3* | c.3321_3329del | W | Occult blood, nephrolithiasis | Het | Occult blood | ND* | N | ND | Occult blood | W | N | W | N |
| 5 | *COL4A4* | c.2377dup | Het | Nephropathy | W | N | ND* | N | Het | Nephropathy | ND* | N | ND | N |
| 6 | *COL4A4* | c.3230del | W | N | Het | Hematuria and bilateral altered renal echogenicity | ND | N | ND | N | Het | Hematuria | W | N |
| 7 | *COL4A4* | c.4423G>T | Het | Hematuria, bilateral altered renal echogenicity, Nutcracker phenomenon | W | N | W | N | Het | Hematuria | ND | N | ND | N |
| 8 | *COL4A5* | c.871C>T | W | N | W | N | ND | N | ND | N | ND | N | ND | N |
| 9 | *COL4A5* | c.1033-1G>C | W | N | W | N | ND | N | ND | N | ND | N | ND | N |
| 10 | *COL4A5* | c.1672G>A | Hemi | Proteinuria, hematuria, mild bilateral enhancement of cortical echo, and bilateral cysts | W | N | W | N | Het | Occult blood | ND | N | ND | N |
| 11 | *COL4A5* | c.1951_1954dup | W | N | W | N | ND | N | ND | N | ND | N | ND | N |
| 12 | *COL4A5* | c.2215C>G | W | N | Het | Intermittent occult blood | ND | N | ND | N | ND | N | ND | N |
| 13 | *COL4A5* | c.2403del | W | N | W | N | ND | N | ND | N | ND | N | ND | N |
| 14 | *COL4A5* | c.2678G>T | W | N | Het | Hematuria | ND | N | ND* | N | W | Hematuria | W | N |
| 15 | *COL4A5* | c.2909G>A | W | N | Het | Occult blood | ND | N | ND | N | W | N | Het | Occult blood |
| 16 | *COL4A5* | c.3454+1G>C | W | N | Het | Occult blood | ND | N | ND* | N | ND* | N | ND | N |
| 17 | *COL4A5* | c.3544G>C | W | N | Het | N | ND | N | ND | N | ND | N | ND | N |
| 18 | *COL4A5* | c.3833G>T | W | N | Het | Occult blood, proteinuria, and renal cyst | ND | N | ND | N | ND* | N | ND* | N |
| 19 | *COL4A5* | c.4730A>C | W | N | W | N | ND | N | ND | N | ND | N | ND | N |
| 20 | *COL4A5* | Exon14_19del | ND | N | ND | Chronic nephritis with hypertension | ND* | N | ND | N | ND | N | ND | N |

Status (S); phenotype (P); heterozygous (Het); hemizygote (Hemi); wildtype (W); not detected (ND); Normal (N); the * denotes deceased individuals.
